# Standardised high dose versus low dose cranberry Proanthocyanidin extracts for the prevention of recurrent urinary tract infection in healthy women [PACCANN]: a double blind randomised controlled trial protocol

**DOI:** 10.1186/s12894-018-0342-7

**Published:** 2018-05-02

**Authors:** Babar Asma, Leblanc Vicky, Dudonne Stephanie, Desjardins Yves, Howell Amy, Dodin Sylvie

**Affiliations:** 1 Department of Obstetrics and Gynaecology, Laval University, CHU de Québec - Université Laval, 2705, boulevard Laurier, Local A1385, Québec, Québec G1V 4G2 Canada; 2 Institute of Nutrition and Functional Foods, Laval University, 2440 Hochelaga Boulevard, Quebec City, Quebec G1V 0A6 Canada; 3 Rutgers University, 125A Lake Oswego Rd., Chatsworth, NJ 08019 USA

**Keywords:** Recurrent urinary tract infection, Women’s health, Proanthocyanidins, Cranberry, *Vaccinium macrocarpon*, Antioxidants, Prevention

## Abstract

**Background:**

Urinary tract infections (UTIs) are amongst the most common bacterial infections affecting women. Although antibiotics are the treatment of choice for UTI, cranberry derived products have been used for many years to prevent UTIs, with limited evidence as to their efficacy. Our objective is to assess the efficacy of a cranberry extract capsule standardized in A-type linkage proanthocyanidins (PACs) for the prevention of recurrent urinary tract infection.

**Methods:**

We will perform a 1:1 randomized, controlled, double blind clinical trial in women aged 18 years or more who present ≥2 UTIs in 6 months or ≥ 3 UTIs in 12 months. One hundred and forty-eight women will be recruited and randomized in two groups to either receive an optimal dose of cranberry extract quantified and standardized in PACs (2 × 18.5 mg PACs per day) or a control dose (2 × 1 mg PACs per day). The primary outcome for the trial is the mean number of new symptomatic UTIs in women during a 6-month intervention period. Secondary outcomes are: (1) To evaluate the mean number of new symptomatic UTIs with pyuria as demonstrated by a positive leucocyte esterase test; (2) To detect the mean number of new symptomatic culture-confirmed UTIs; (3) To quantify urinary PACs metabolites in women who take a daily dose of 37 mg PACs per day compared to women who take a daily dose of 2 mg per day for 6 months; (4) To characterize women who present recurrent UTI based on known risk factors for recurrent UTI; (5) To describe the side effects of daily intake of cranberry extract containing 37 mg PACs compared to 2 mg PACs. This report provides comprehensive methodological data for this randomized controlled trial.

**Discussion:**

The results of this trial will inform urologists, gynaecologists, family physicians and other healthcare professionals caring for healthy women with recurrent UTI, as to the benefits of daily use of an optimal dose of cranberry extract for the prevention of recurrent UTI.

**Trial registration:**

Clinicaltrials.gov, identifier: NCT02572895 October 8th 2015.

## Background

Urinary tract infections (UTIs) are one of the most common bacterial infections affecting women [1, 2]. UTI preferentially affects young, sexually active women with 50–60% of women reporting at least one UTI during their lifetime [2]. Nearly 1 in 3 women will experience at least one episode of UTI requiring antibiotic therapy before the age of 24 years and a quarter of these women will present reoccurrence within 6 months [1]^.^ Anatomical differences in male and female perineal anatomy may explain why women are more susceptible than men to the ascension of faecal bacteria in the urinary tract. More precisely, these anatomical features include a relative shortness of the urethra [3], the urethral meatus’s proximity to the anus and a more humid surrounding environment comparatively to the male anatomy [4, 5]. Additional well-established risk factors include spermicide-based contraception, history of previous UTI, first UTI before 15 years of age and those with UTI history in the mother [6–10].

Recurrent UTIs (r-UTI) are defined as more than 2 episodes in the last 6 months or 3 episodes in the last year [11]. The major symptoms of UTI include dysuria, increased frequency of urine, cloudy urine and occasionally hematuria [12]. In general, uncomplicated UTIs are confined to the bladder, resolve quickly following antibiotic treatment and thus are associated with fewer severe or long-term sequelae [13]. Though viewed as a benign affliction, uncomplicated lower UTI symptoms can have considerable impacts on the patient’s productivity and quality of life. A study of university women reported that patients suffering from UTI experienced 2.4 days of restricted activity, 1.2 days of lost time and 0.4 bed bound days due to their symptoms [7]. Presently, the Canadian Urological Association and Society of Obstetricians and Gynecologists of Canada recommendations for the treatment of uncomplicated r-UTI use one of three antibiotic treatment regimens: continuous antibiotic prophylaxis, post-coital antibiotic prophylaxis or self-start antibiotic therapy [11, 12]. Empirical and preventive antibiotics for the treatment of r-UTI have been established as the most cost-effective way to manage these infections. However, prescribing without confirmation of diagnosis and isolation of causal bacterial contributes to the growing problem of uropathogen resistance in primary care [14].

Cranberries have been used for numerous years to prevent UTIs. Research suggests that proanthocyanidins (PACs), a component of cranberries, inhibit the adherence of p-fimbriated *Escherichia coli* on uroepithelial cells of the bladder, preventing the adherence of bacteria to the mucosal surface of the urinary tract and thereby inhibiting bacterial proliferation [15]. A multicentre randomized clinical trial (RCT) in sexually active adult women showed that a daily dose of 36 mg PACs or more provided an optimal antibacterial effect in the urine [16]. A Cochrane systematic review published in 2012, could not definitively conclude on the efficacy of cranberry products for the prevention of r-UTI mainly because of a lack of observance to the intake of cranberry supplements in juice form and also because of varying PACs concentrations in the different clinical trials that were rarely quantified or standardized [17]. A systematic review in 2013 including trials conducted in women with r-UTI found a significantly decreased risk of UTI among women receiving cranberry-containing products compared to control (2 trials, Risk ratio (RR) = 0.53, 95%CI 0.33–0.83, I^2^ = 0%) [18].

Since these reviews, several well-designed trials have evaluated the effects of daily cranberry capsules in women with r-UTI. Most notably, an RCT compared the preventive effects of a cranberry juice (UR65) containing 40 mg PACs versus placebo on the relapse of UTI in women aged 20–79 years presenting acute complicated or uncomplicated cystitis with a history of r-UTI during a six-month follow-up [19] The authors found no significant difference in the relapse rates of UTI between groups (log-rank test, *p* = 0.4209). Similarly, a double blind RCT evaluated the effects of a daily dose of 500 mg of a commercial cranberry fruit powder (2 mg PACs) compared to a placebo in 176 women who experienced at least 2 symptomatic UTI in the twelve months preceding the study [20]. In this trial, the proportion of women who experienced greater than one UTI in the cranberry powder group was significantly lower than in the placebo group during the six-month follow-up period (10.8% vs 25.8%, *p* = 0.04). Although well designed, these studies presented several methodological flaws such as the inclusion of women with complicated UTIs related to catheterization in the former as well as the use of a sub-optimal dose of cranberries in the latter.

We hypothesize that the efficacy of cranberry products for the prevention of R-UTI in women will be strongly increased by the usage of an optimal dose of cranberry extracts in capsule form (standardized to 37 mg PACs per day) and by an adequate measure of participant observance.

## Methods

### Study design and objectives

**Fig. 1:**
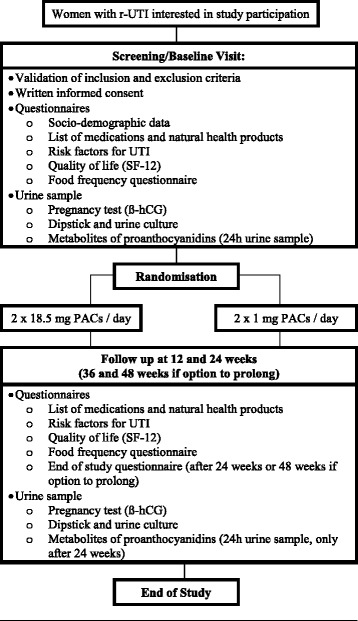
Study procedures and characteristics of study visits

To assess the effects of a standardized cranberry extract in sexually active healthy women who present r-UTI, we will undertake a double blind, prospective RCT with 2 arms comparing the mean number of new UTIs during a 6-month period after consumption of a standardized cranberry extract containing 37 mg PACs (2 × 18.5 mg PACs per day) with a control dose of 2 mg PACs (2 × 1 mg PACs per day) in women presenting r-UTI. This protocol was developed in accordance with the Standard Protocol Items: Recommendations for Interventional Trials (SPIRIT) Statement. The SPIRIT figure is illustrated in Fig. 1.

The primary objective is to evaluate, in sexually active women who present r-UTIs, the effects of a standardized cranberry extract containing 37 mg type-A linkage PACs per day, compared to a control dose of 2 mg PACs per day during a 6-month period on the incidence rate of newly symptomatic UTIs during a 6-month follow-up period. Secondary objectives are: (1) To evaluate the mean number of new symptomatic UTIs with pyuria as demonstrated by a positive leucocyte esterase test; (2) To detect the mean number of new symptomatic culture-confirmed UTIs; (3) To quantify urinary PACs metabolites in women who take a daily dose of 37 mg PACs per day compared to women who take a daily dose of 2 mg per day for 6 months; (4) To characterize women who present r-UTI based on known risk factors such as spermicidal contraception use, frequency of sexual relations, personal and familial history of UTI; (5) To describe the side effects of daily intake of cranberry extract containing 37 mg PACs compared to 2 mg PACs.

### Study participants and recruitment

This clinical trial aims to enrol sexually active non-pregnant non-lactating women aged 18 years and over presenting culture-confirmed r-UTI (defined as ≥2 UTIs in the past 6 months or ≥ 3 UTIs in the past 12 months). Women will be recruited in the Laval University community in Quebec City, Canada, through list serves and local clinician referrals as well as posters in medical clinics, social media, paid advertising and word of mouth. Women wishing to participate will contact the study coordinator who will explain the research project to them and verify eligibility according to inclusion and exclusion criteria (Table 1). The risks and benefits of the study will be thoroughly discussed and the consent form will be signed at the first of three visits at the Institute on Nutrition and Functional Foods (INAF).

**Table 1:** Admissibility criteria for the cranberry extract for prevention of recurrent urinary tract infections trial

Inclusion Criteria	
Sexually active healthy women	
Aged 18 years and older	
Recent history of recurrent urinary tract infections (UTIs)^a^	
≥ 2 UTIs in the past 6 months and/or	
≥ 3 UTIs in the past 12 months	
No consumption of cranberry juice, polyphenol or antioxidant supplements in the last 2 weeks	
Exclusion Criteria	
Pregnancy	
History of anatomical urogenital anomalies, urogenital tract surgery	
History of acute or chronic renal failure, nephrolithiasis	
History of intestinal diseases causing malabsorption	
Anticoagulant medication in the last month	
Known allergy or intolerance to cranberry	

^a^UTIs diagnosed by a clinician and treated with antibiotic therapy

Potential participants will need to restrain exposure to systemic antimicrobial agents or cranberry derivatives in the two weeks preceding enrolment. Women with anatomical abnormalities of the urinary tract, a history of renal disease (renal failure, nephrolithiasis) or intestinal disease causing malabsorption (Crohn’s disease, Celiac disease), or anticoagulant therapy will be excluded. Furthermore, we will exclude women with known allergy or intolerance to cranberries.

#### Study randomization

Concealed randomization will be generated using computer aided block randomization by blocks of 10. Eligible women will be assigned 1:1 to consume a cranberry extract either formulated in high PAC content capsules (2 capsules of 18.5 mg PAC per day) or low PAC content capsules (2 capsules of 1 mg PAC per day) for 6 months. The low PAC content cranberry capsule is comparable to the majority of cranberry extract products presently approved by Health Canada [21] . The standardized capsules will be manufactured and provided by Diana Foods and will be distributed in opaque packaging in order to conceal colour variations from the research team. The PAC content of each cranberry treatment will be validated at INAF according to the standardized BL-DMAC method, using A2 procyanidin dimer as standard for the quantification [22]. All clinical investigation, laboratory analysis, data collection and assessment will be blinded to the randomization allocation.

#### Clinical follow-up

Each visit (0, 12 and 24 weeks) will include a short questionnaire documenting socio-demographic characteristics (*T* = 0 only), medication and natural health product intake, quality of life (SF-12) [23], risk factors for UTIs, and a validated food frequency questionnaire (FFQ) [24] modified for our study to specifically include foods containing PACs. Double data entry will be performed under the study coordinator supervision in order to promote data quality. Participants will be instructed to obtain a midstream urine sample according to standard methods suggested by the microbiological laboratory. A dipstick urinalysis using Chemstrip 9 (Roche Diagnostics USA) and a pregnancy test (ß-hCG) will be completed. Three 10 ml tubes will be placed in a − 80 °C freezer for urinary PAC metabolites measurements by ultra-high performance liquid chromatography coupled with mass spectrometry, and the urine sample for culture will be refrigerated and transported to the microbiology laboratory at the CHU de Quebec – Universite Laval within 24 h after collection.

During their participation, the women will be asked to contact the study coordinator if they present symptoms of UTI in order to schedule a visit at INAF to confirm the diagnosis by urinalysis and urine culture and to receive an appropriate antibiotic prescription based on their medical history and allergies. Women unable to present themselves at INAF will be prescribed antibiotics waiving a confirmation of their UTI. Women wishing to discontinue the consumption of capsules will be asked to present themselves at the 3 and 6-month visit in order to complete intention to treat analysis. The participants will be asked not to consume other products containing cranberry derivatives for the duration of the study and to consume their cranberry extracts 2–4 h preceding each visit. Participants will also be asked to limit intense physical activity for 24 h preceding each visit. After 24 weeks of participation, women will be provided with the option of prolonging their participation for an additional 24 weeks, with follow-up visits at 36 and 48 weeks. This data will provide information on seasonal variations in the incidence of UTI.

#### Sample size and statistical analysis

Statistical analyses will be performed using SAS 9.2 (SAS Institute Inc., USA). All analyses will be based on the intention-to-treat principle before unmasking the treatment groups. The baseline characteristics of both groups will be compared using a Student’s t test for continuous variables and a generalized linear model for nominal variables. The Poisson regression model, which allows us to adjust for participants that were lost to follow up, will be used to compare the incidence of UTI during the 6-month follow up period as well as side effects of the treatment. Subgroup analyses will be performed in order to evaluate the impact of cranberry capsules in pre- and post-menopausal women as well as women with certain risk factors for complicated UTI such as pelvic floor disorders and diabetes.

Based on the literature [17], we estimate that 35% of patients in the control group will present at least one UTI during the 6-month follow-up period. In total, 126 women will need to be recruited in order to detect a clinically significant difference of 25% between the 2 groups (10% of women assigned to the experimental group will have at least 1 UTI with a power of 80%). Based on our past clinical trial experience [25], we estimate that 15% of randomized participants will be lost to follow up, therefore 148 women will need to be recruited in order to have at least 126 participants who will complete the 24-week intervention.

## End points

### Incidence of UTI

The primary endpoint is the average number of symptomatic UTIs during the 6-month follow-up period. Individuals with acute urinary symptoms such as pollakiuria, urgency, burning, suprapubic pain, and hematuria will be assessed by study staff and will have to provide a urine sample for urinalysis. Women who present both symptom and pyuria criteria, defined as a positive leukocyte esterase dipstick result, will be diagnosed as having confirmed symptomatic UTI and prescribed appropriate antibiotic treatment. If the urine culture results are positive, the episode will be categorized as a culture-confirmed UTI. Women with symptomatic UTI during the study period will continue to take the cranberry capsules and remain in the study for the full 6 months unless they are lost to follow up or discontinue the intervention.

### Urinary PAC content

At the beginning of the trial, participants will be offered the option to provide a 24-h urine collect for targeted metabolomic characterization of PAC metabolites using ultra-high-performance liquid chromatography coupled to tandem mass spectrometry, performed by a chemist blinded to treatment allocation.

### Compliance and side effects

The cranberry extract capsules will be distributed at each visit and participants will be asked to bring remaining capsules the next visit in order to count remaining capsules. The participants will fill out a daily journal to record compliance, transient UTI symptoms and any adverse effects related to capsule intake. A bi-monthly email reminder will be sent to encourage participation. Each participant will receive an email reminder in the week preceding each visit. Evaluation of side effects will take place at each visit and participants will be asked to document symptoms (nausea, dyspepsia, abdominal pain, bloating and headaches) in their daily journal. In the presence of severe side effects, participants will be allowed to discontinue the intervention and remain in the study in order to preserve the intention to treat analyses.

### Blinding and contamination Bias

The proportion of women who will guess their group allocation correctly will be documented with a short questionnaire at the last visit. To control for contamination bias, any antibiotic therapy during the study period will be declared to the study coordinator and PAC consumption will be measured by FFQ for the 24 h preceding each visit.

## Discussion

Use of cranberry derived products in the prevention of r-UTI remains controversial, with no definitive evidence to show superiority of the cranberry compared to antibiotic therapy [17, 18]. There is some evidence that cranberry products may reduce the incidence of UTIs compared to placebo, though the most effective amount and concentration of PACs that must be consumed and the duration for the intervention are unknown [17]. To our knowledge, this study is the first large, prospective randomized clinical trial assessing the impact of a cranberry extract capsule standardized to 37 mg PAC per day compared to 2 mg PAC per day in preventing UTIs in healthy women presenting r-UTI.

Incidence of symptomatic UTI during the 6-month follow-up period was selected as the primary end-point in this trial because it is the most important, clinically relevant long-term outcome for patients. We used three classifications for UTI analyses: symptomatic UTI, dipstick-positive UTI and culture-confirmed UTI. Symptomatic UTI was diagnosed if a participant presented at least one of the following symptoms: dysuria, pollakiuria, urinary urgency, suprapubic pain or hematuria. In the presence of clinical symptoms, participants will be asked to provide a urine sample in order to perform a dipstick test and a urine culture. Positive leucocytes or nitrites will indicate a positive dipstick test [26]. A positive urine culture will be designated by greater than 10^6^ colony-forming units, according to the microbiological laboratory standards of the CHUL hospital. In our study, antibiotics will be prescribed in the presence of clinical symptoms in combination with either a positive urine dipstick test and/or positive urine culture.

### Implications of findings

Many Canadian women who present r-UTI commonly use over the counter cranberries-derived products with inadequate labelling of PACs concentrations. For cranberry products with quantification of PACs, these concentrations rarely exceed 2 mg in Canada [21]. Various trials have tested the effectiveness of cranberry derived products, essentially in juice form, and their results remain discordant mainly due to the lack of standardization and low doses of PACs in tested products. Hence, the intrinsic activity of cranberry PACs, demonstrated against *Escherichia coli* in vitro, has never been optimized for the prevention of UTI. The results generated from this trial will clarify the role of cranberry extracts standardized in PACs on the decreased incidence of UTI in women presenting r-UTIs, will evaluate the differences in the incidence of UTI on the basis of different PACs concentrations and will respond to the recommendations reported in the last Cochrane meta-analysis [17].

This report provides comprehensive methods for a clinical trial on the prevention of RUTI by cranberry extract capsule intake. The strengths of this trial include the quantification and standardisation of PACs contained in the cranberry extract capsule and a randomized, double blind, controlled trial method. Our trial will add to a growing body of literature regarding cranberry extract capsule for the prevention of r-UTI in healthy women. In addition, the data set and specimen bank generated from conducting this trial will enable researchers to understand the metabolites of type-A PACs produced after prolonged consumption of cranberry capsules.

## Trial status

Participant recruitment started on August 18th 2015 and was completed in March 2017. Study follow up visits will continue into Winter 2018.

## Abbreviations

FFQ: Food frequency questionnaire
PAC: Proanthocyanidin
RCT: Randomized controlled trial
r-UTI: Recurrent UTI
UTI: Urinary tract infection
